# Identification of microflora related to growth performance in pigs based on 16S rRNA sequence analyses

**DOI:** 10.1186/s13568-020-01130-3

**Published:** 2020-10-29

**Authors:** Xin-Jian Li, Mingyu Wang, Yahui Xue, Dongdong Duan, Cong Li, Xuelei Han, Kejun Wang, Ruimin Qiao, Xiu-Ling Li

**Affiliations:** College of Animal Science and Technology, Henan Agricultural University, Zhengzhou, 450046 China

**Keywords:** 16S rRNA gene, Gut microbiome, Growth performance, Pig

## Abstract

Intestinal microorganisms have been shown to be important factors affecting the growth performance of pigs. Therefore, to investigate the effect of the intestinal microflora structure on the growth performance of pigs, samples from Duroc (n = 10), Landrace (n = 9) and Yorkshire (n = 21) pigs under the same diet and feeding conditions were collected. The fecal microbial composition was profiled via 16S ribosomal RNA (rRNA) gene sequencing. We also analyzed their growth performance. We found that Duroc and Landrace pigs had significant differences in average daily gain (ADG), feed efficiency ratio (FER), growth index (GI), and number of days taken to reach 100 kg (*P* < *0.05*). Moreover, through analysis of the intestinal flora, we also identified 18 species of intestinal flora with significant differences between Duroc and Landrace pigs (*P* < *0.05*). To eliminate the influence of genetic background, the differential intestinal flora of 21 Yorkshire pigs with differences in growth performance was analyzed. The results showed that there were significant correlations between *Barnesiella*, *Dorea*, *Clostridium* and *Lactobacillus* and pig growth performance. To explore the effect of the intestinal flora on the growth performance of pigs at the molecular level, *Lactobacillus*, which is the most abundant in the intestine, was selected for isolation and purification and cocultured with intestinal epithelial cells. qPCR was used to determine the effect of *Lactobacillus* on MC4R gene expression in intestinal epithelial cells. The results showed that *Lactobacillus* inhibited MC4R gene expression in these cells. The results provide a useful reference for further study of the relationship between the intestinal flora and pig growth performance.

## Introduction

There are a large number and a wide variety of symbiotic bacteria living in the intestines of animals. The number of microbes in the intestines of humans and animals is up to 10^14^, nearly 10 times the number of animal body cells, and the mass can be as high as 1.2 kg, which is close to the mass of the human liver. These microorganisms include bacteria, archaea, viruses and fungi, among which bacteria are the most numerous (Chen et al. 2015; Uyeno et al. 2015). The intestinal flora can provide nutrients and energy for the body, regulate immunity, antagonize pathogenic microorganisms, participate in metabolism, and even affect host behavior (Collins et al. 2012; Kim and Isaacson 2016).

With the popularization of low-cost "next-generation sequencing" technology that yields large data sets, researchers have studied the microbial communities in soil, ocean, fresh water, air and other natural environments and discovered many unknown microorganisms, deepening their understanding of microbial diversity in nature (Kim and Isaacson 2015). Pig gut microbes are mainly distributed in the cecum, and the number of microorganisms in the intestinal contents (per gram) is 10^12^–10^13^ colony-forming units (CFU), composed of 400–500 kinds of microbes, mainly Bacteroides species (8.5–27.7%) and the thick-walled *Clostridium XIV* group (10.8–29.0%), with the *Clostridium IV* group (25.2%) constituting the advantageous bacterium group (Leser et al. 2002).

The growth performance of animals is closely related to economic benefits, and improving the growth performance of animals is an important research direction in the breeding industry. Studies have shown that gut microbes are also involved in regulating animal growth. Xin et al. (2009) found that *Lactobacillus johnsonii* BS15 could significantly improve the daily weight gain and diarrhea index of piglets, and improve the growth and development ability and disease resistance of piglets to a certain extent. Sato et al. (2019) found that adding *Enterobacter faecalis* to the diet of weaned piglets can effectively improve the growth performance of pigs, and adding *Enterococcus faecalis* and *Clostridium butyricum* to the diet may have certain effects on the structure of the intestinal flora. Niu et al. (2019) found that the bacterial abundance of *Clostridium* and *Turicibacter* species in sow intestines was positively correlated with the apparent digestibility of ether extract, and that of *Anaerofustis* and *Robinsoniella* was positively correlated with the apparent digestibility of crude fiber. The abundance of *Collinsella* and *Sutterella* and the apparent digestibility of neutral detergent fiber were positively correlated (Yang et al. 2019; Sato et al. 2019). Li et al. (2019) used Yorkshire pigs to study the function of gut microbes and found that gut microbes can improve nutrient digestibility for fattening pig growth and the regulation of volatile fatty acids.

However, different pig breeds have characteristic intestinal microbes, and the widely used Duroc, Yorkshire and Landrace pigs exhibit differences in growth traits. To date, the screening of growth-related microorganisms by comparing the differences in intestinal microorganisms among the three breeds has not been reported. Therefore, in order to analyze the differences in the intestinal flora of different breeds of pigs and screen out intestinal flora related to the growth performance of pigs, this study first analyzed the intestinal flora of Duroc, Yorkshire and Landrace pigs, and the relation of pig growth performance with the intestinal flora was determined by preliminary screening. To eliminate the influence of genetic background, this study used selected key flora of the Yorkshire pigs, and correlation analysis of pig growth performance was carried out to screen the intestinal flora. Finally, the function of the selected key flora was verified at the cellular level. The purpose of this study was to screen out the key microflora related to the growth performance of pigs through the above studies and to preliminarily explore their functions. This study lays a foundation for improving the scientific knowledge of the regulation of economically important characteristics of pigs by the intestinal flora.

## Materials and methods

### Animals and growth performance measurements

First, we explored and identified the diversity of the intestinal flora in pig intestines and the key intestinal flora related to pig growth performance in forty breeding boars (Duroc, n = 10; Landrace, n = 9; and Yorkshire, n = 21) with an average body weight (BW) of 97.97 ± 2.88 kg. Second, to eliminate the influence of genetic background, twenty-one Yorkshire pigs with an average BW of 96.62 ± 4.20 kg were collected. The three breeds were fed the same diet based on corn and soybean, grown under the same hog pen, and housed in comfortable temperature and humidity conditions.

The BW of each animal was recorded after the animal reached approximately 20 kg and at the end of the experiment three months later. Body measurement traits, including body length (BL), body height (BH), chest girth (CG), rump girth (RG), tube girth (TG) and backfat thickness (BT), were measured at the end of the experimental period. The values for the average daily gain (ADG), feed efficiency ratio (FER), growth index (GI), and number of days required to reach 100 kg were provided by the staff in the feedlot and were compiled. All the measured data were corrected and analyzed by SPSS 22.0.

### Sample collection and 16S rRNA gene sequencing

Fecal samples were collected from all pigs via rectal massage at the end of the experimental period and stored in liquid nitrogen. Total genomic DNA was extracted using the QIAamp DNA Stool Mini Kit (Qiagen, Hamburg, Germany) according to the manufacturer’s instructions (Yang et al. 2014). The concentration and quantity of the DNA were measured using a NanoDrop 1000 spectrophotometer (NanoDrop, Wilmington, DE), and the DNA was diluted to 1 ng/μL using sterile water.

16S ribosomal RNA (rRNA) gene sequencing was performed by Shanghai Sangon Biotech on Illumina HiSeq 2500. The distinct V3-V4 regions of the 16S rRNA genes were amplified using specific primers (forward: GTGCCAGCMGCCGCGGTAA and reverse: GGACTACHVGGGTWTCTAAT, with barcodes).

Polymerase chain reactions (PCRs) were performed in triplicate in a total volume of 30 μL, containing 4 μL of primers, 30 ng of DNA template, 25 μL of PCR Master Mix and molecular-biology-grade water as needed. The following PCR thermocycling conditions were used: an initial denaturation at 98 °C for 3 min, followed by 30 cycles of 98 °C for 45 s, 55 °C for 45 s, and 72 °C for 45 s, with a final extension at 72 °C for 7 min. The PCR products were purified using Agencourt Ampure XP beads (Beckman Coulter, Inc.) and used to construct libraries. Finally, 24 samples subjected to 250-bp paired-end sequencing on the Illumina HiSeq/MiSeq platform (Illumina, United States) at Shanghai Sangon Biotech.

### Sequence filtering and taxonomic assignments

The Illumina MiSeq raw image data were transformed by CASAVA base recognition analysis into the original sequences, known as raw data or raw reads, and the results were stored in FASTQ format. After removing the primer and adapter sequences, the paired reads were merged into single sequences according to the overlap between the paired-end reads. Then, the samples were identified and distinguished according to the barcode sequences to obtain the sample data. Finally, the data for each sample were quality-controlled and filtered to obtain valid data for each sample. USEARCH was used to remove the unamplified sequence regions of the pretreated sequence, after which sequencing errors were corrected (Edgar et al. 2010), and UCHIME was used to identify the chimeras (Edgaret al. 2011). Subsequently, we performed BLASTn comparisons for the deleted chimeric sequences and representative database sequences. The alignment results below a specific threshold were considered to be sequences outside the target region, and the partial sequences were removed.

### Statistical analysis

Community diversity within and between groups was assessed using several indices, including the observed species, Chao1 estimator, abundance-based coverage estimation (ACE), Shannon and Simpson indices, all of which were calculated using mothur (Schloss et al., 2009). The Wilcoxon rank-sum test was used to measure the differences in α-diversity values among the three groups, with *P* < *0.05* considered significant. Using the results of the taxonomic analysis, taxonomic comparisons between one or more samples at each classification level can be obtained. Correlation analysis is a classic method used to analyze the interactions between microorganisms. During the analysis, species or optical transform units (OTUs) with an abundance of more than 1% or with an abundance ranking in the top 100 were selected for the bilateral test. SparCC (Friedman et al. 2012) was used to calculate the correlation coefficient and p value between each community/OTU, and the corrplot package (Wei et al., 2017) in R was used to plot the correlation matrix graph.

Phylogenetic measurements of β-diversity were also estimated using QIIME (Kuczynski et al., 2011). The unweighted UniFrac distance was used for principal coordinate analysis (PCoA) to compare the microbial communities from the three groups. Illustrations were generated using the vegan package in R. Linear discriminant analysis effect size (LEfSe*)* was used for the discovery and interpretation of biological markers and characteristics at multiple levels. This analysis uses statistical methods to assess different characteristics of the discovery and significance tests, where the program first uses the nonparametric coefficient Kruskal–Wallis (KW) rank-sum test to detect the abundances and characteristics of the significant differences between groups to identify any association between groups of subgroups. Subsequently, a Wilcoxon rank-sum test (unpaired) is used to assess the differences in the features of the group through a consistency check, after which LDA is performed to estimate the influence of the differences in group size. PICRUSt (Langille et al. 2013) was used to predict functional enrichment from the 16S rRNA gene sequencing data with the Greengenes database. The significant differences between pairs of samples or multiple groups of Kyoto Encyclopedia of Genes and Genomes (KEGG) and Gene Ontology (GO) pathways were measured using STAMP (Parks et al. 2014).

### Cell culture and isolation and purification of bacteria

Lactobacilli were isolated from fecal samples according to the test methods of Mirelahi et al. (2009). Then, the lactic acid bacteria were cultured, and bacterial suspensions was prepared at 10^6^, 10^7^ and 10^8^ CFU. The cells were stored at 4 °C and set aside. Subsequently, pig intestinal epithelial cells were isolated and cultured according to the method of Deguchi et al. (2006). After cultivation for 24 h, the cells were treated with different concentrations of the bacterial suspension prepared in the previous stage. The cells were collected after being cocultured with the bacterial suspension for 12 h, and RNA was extracted from the cells according to the instructions for the RNA extraction kit used. The concentration and quantity of RNA were measured using a NanoDrop 1000 spectrophotometer (NanoDrop, Wilmington, DE), and the qualifying RNA samples were then stored in a refrigerator at a low temperature for later use. A number of studies have shown that there is a significant correlation between the expression of the MC4R gene and growth performance, so the MC4R gene is considered an important candidate gene for regulating growth performance (Kim et al. 2000). Based on the above research, the MC4R gene was selected as the candidate gene in this study to study the influence of the intestinal flora on gene expression. A kit from Takara was used for reverse transcription and qPCR to detect the expression level of the MC4R gene. CFX_3StepAmp + Melt was used for qPCR. HMBS was selected as the internal reference gene.

## Results

### Growth performance analysis of the three breeds of pigs

**Fig. 1:**
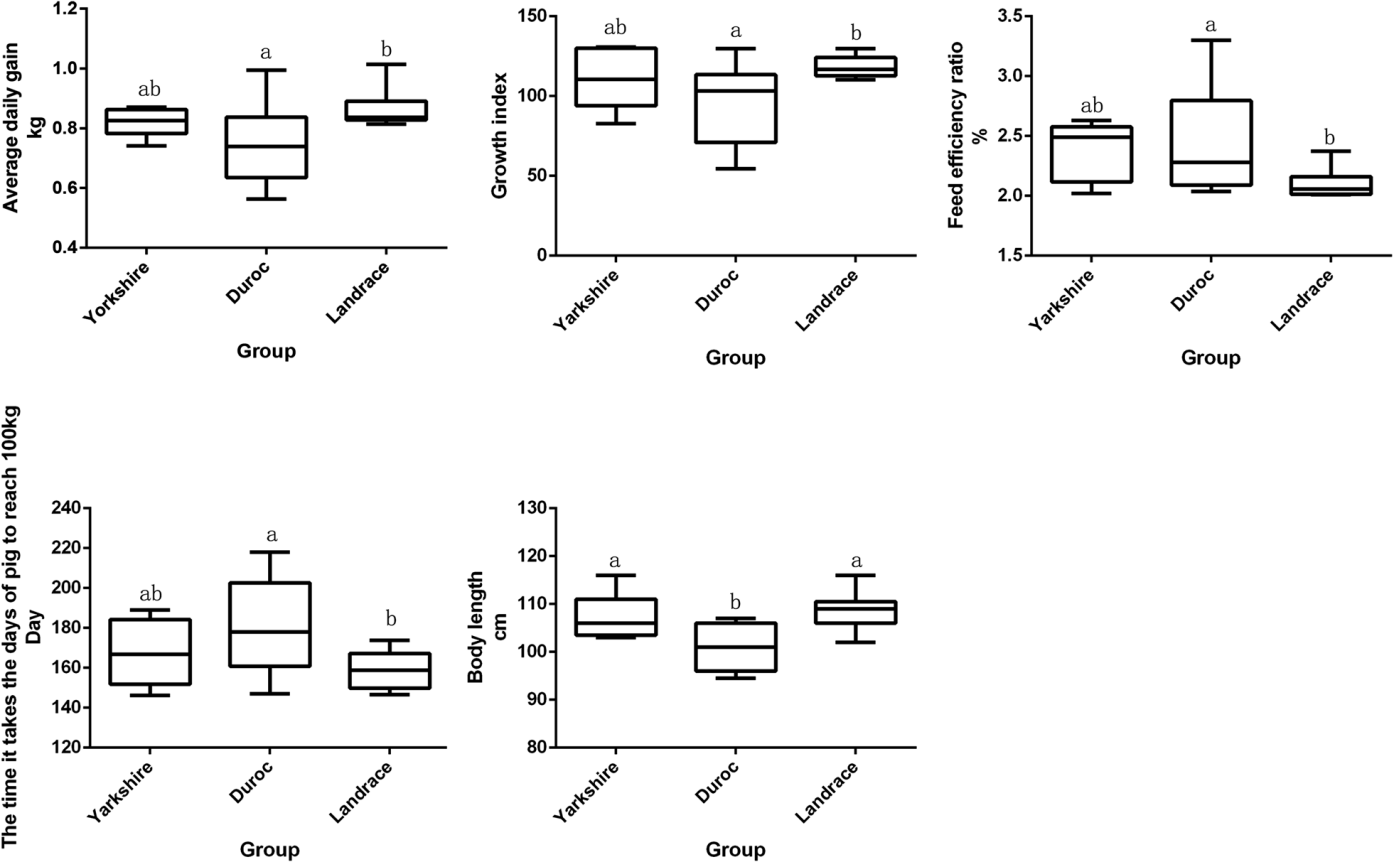
Distribution of the growth traits of the boars of the three pig breeds. The error bars represent standard deviations. The middle line in the bar represents the median. In the same set of data, different superscript letters (ab) indicate a significant difference (*P* < 0.05)

The growth performance of the three different pig breeds was compared, and the results showed that the GI (96.42), ADG (0.746 kg) and BL (101.1 cm) of Duroc pigs were significantly lower than those of Landrace pigs (GI = 118.17, *P* = 0.021; ADG = 0.864 kg, *P* = 0.014; and BL = 108.8 cm, *P* = 0.027, respectively) (Fig. 1). These three traits of Duroc pigs were also lower than those of Yorkshire pigs, although the differences were not significant (*P* > 0.05). In contrast, the FER (2.489) and the days required to reach 100 kg (180.5 days) for Duroc pigs were significantly higher than those observed for Landrace pigs (Fig. 1). For the other indicators, no significant differences among the three breeds were observed (Additional file 1: Table S1).

### Bacterial diversity and composition in the three pig breeds

A total of 160,497 valid sequence reads were generated, after which OTUs with a 97% identity cutoff were identified in the Duroc (1,222), Landrace (1,372), and Yorkshire (1,311) pigs (Additional file 2: Table S2). Yorkshire pigs exhibited the highest Shannon diversity index (4.19) and had a more diverse bacterial community than the Landrace (4.13) and Duroc (3.75) pigs. The ACE and Chao1 values observed for Duroc pigs (2,704.33 and 2,118.08, respectively) were lower than those observed for Landrace (3,027.20 and 2,348.21, respectively) and Yorkshire (2,725.23 and 2,170.14, respectively) pigs, showing that the richness of gut microbial species in Duroc pigs was lower than that in the other two breeds. In addition, the coverage for the three breeds was more than 98.8%, suggesting that most of the fecal bacterial diversity was captured (Additional file 2: Table S2).

**Fig. 2:**
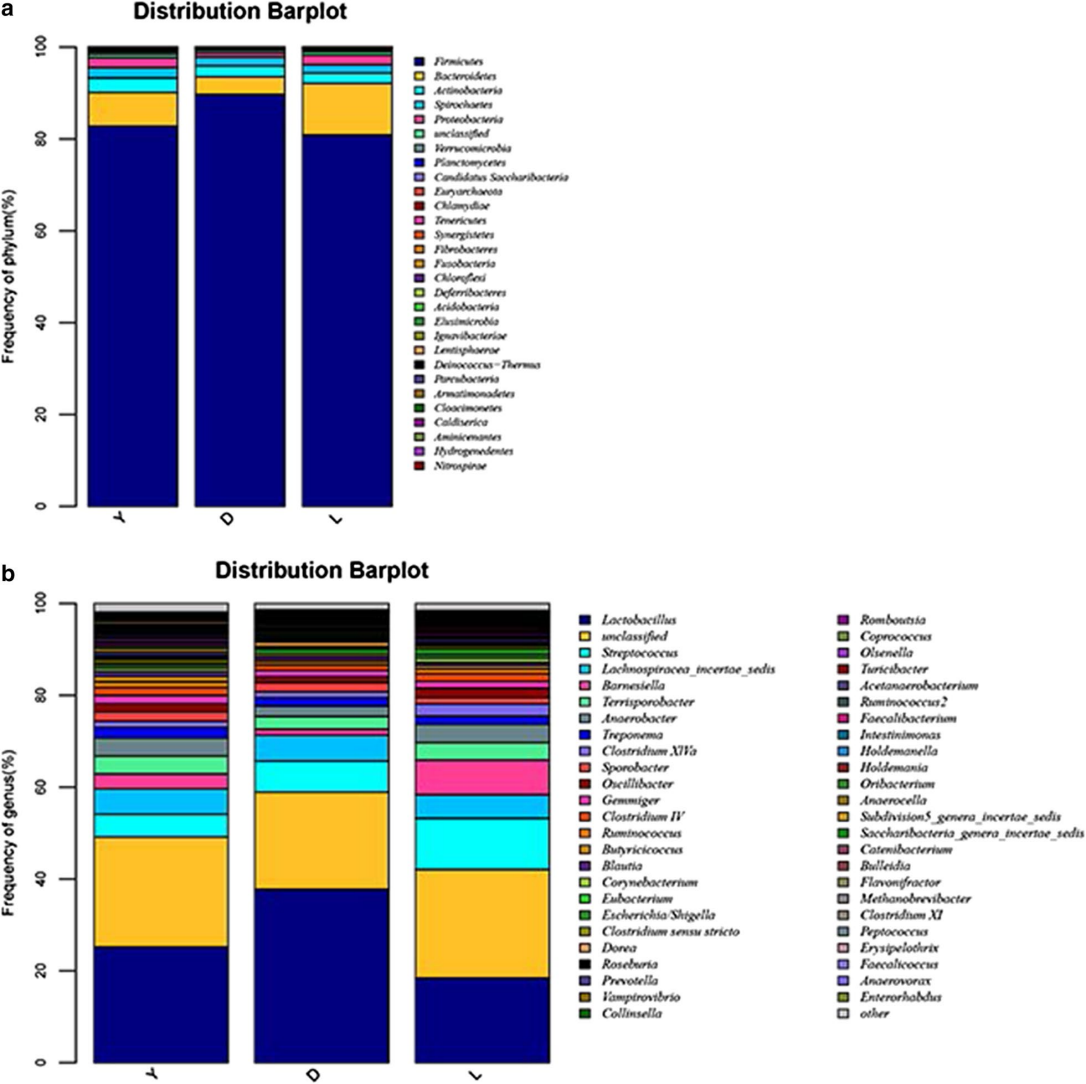
Distribution of bacterial phyla (**a**) and genera (**b**) and their abundances in the fecal microbiota of boars of the three pig breeds. *D* duroc, *L* landrace, *Y* yorkshire

At the phylum level, *Firmicutes* and *Bacteroidetes* dominated the fecal microbiota regardless of breed, with other phyla including *Actinobacteria*, *Spirochetes*, *Proteobacteria*, *Planctomycetes*, *Fibrobacteres*, *Euryarchaeota*, *Verrucomicrobia*, *Chlamydiae* and *Tenericutes*. However, the bacterial community compositions of the breeds differed. For example, the proportion of the phylum *Firmicutes* was greater in the feces of Duroc pigs (89.72%) than in the feces of Landrace (81.13%) and Yorkshire (82.6%) pigs (*P* = 0.02 and 0.11, respectively). In contrast, the proportion of the phylum *Bacteroidetes* was higher in the feces of Landrace (11.03%) and Yorkshire (7.36%) pigs than in the feces of Duroc pigs (3.85%) (*P* = 0.03 and 0.15, respectively). Similarly, the proportion of the phylum *Proteobacteria* was higher in the feces of Landrace (2.06%) and Yorkshire (2.25%) pigs than in the feces of Duroc pigs (0.86%) (p = 0.01 and 0.007, respectively). Finally, the proportion of the phylum *Synergistetes* was greater in the feces of Landrace pigs than in the feces of Yorkshire pigs (*P* = 0.018) (Fig. 2a and Additional file 3: Table S3).

At the genus level, *Lactobacillus*, *Streptococcus*, *Lachnospiracea* and *Barnestella* dominated the fecal microbiota regardless of breed, with other abundant genera including *Terrisporobacter*, *Anaerobacter*, *Treponema*, *Sporobacter*, *Oscillibacter*, *Gemmiger* and *Clostridium*. Although the composition of the intestinal microbiota of the three breeds of pigs was similar, the abundance and proportion of each taxon differed to some extent, with Duroc and Landrace showing the greatest differences among the three breeds (Fig. 2b).

### Identification and functional prediction of key intestinal flora that affect pig growth performance

The PCoA results showed that the microbiomes of the Landrace pigs were clearly separated from those of the Duroc pigs. Clustered samples indicate a high species composition similarity compared to separated samples. The Duroc samples were primarily concentrated in the yellow area at the top of the figure, whereas the Landrace samples were primarily concentrated in the light blue area at the bottom of the figure (Additional file 4: Fig. S1). Most of the OTUs were shared among the three breeds (2082), but 1243, 1455 and 730 OTUs were specifically observed in the Duroc, Landrace and Yorkshire breeds, respectively (Additional file 5: Fig. S2).

**Fig. 3:**
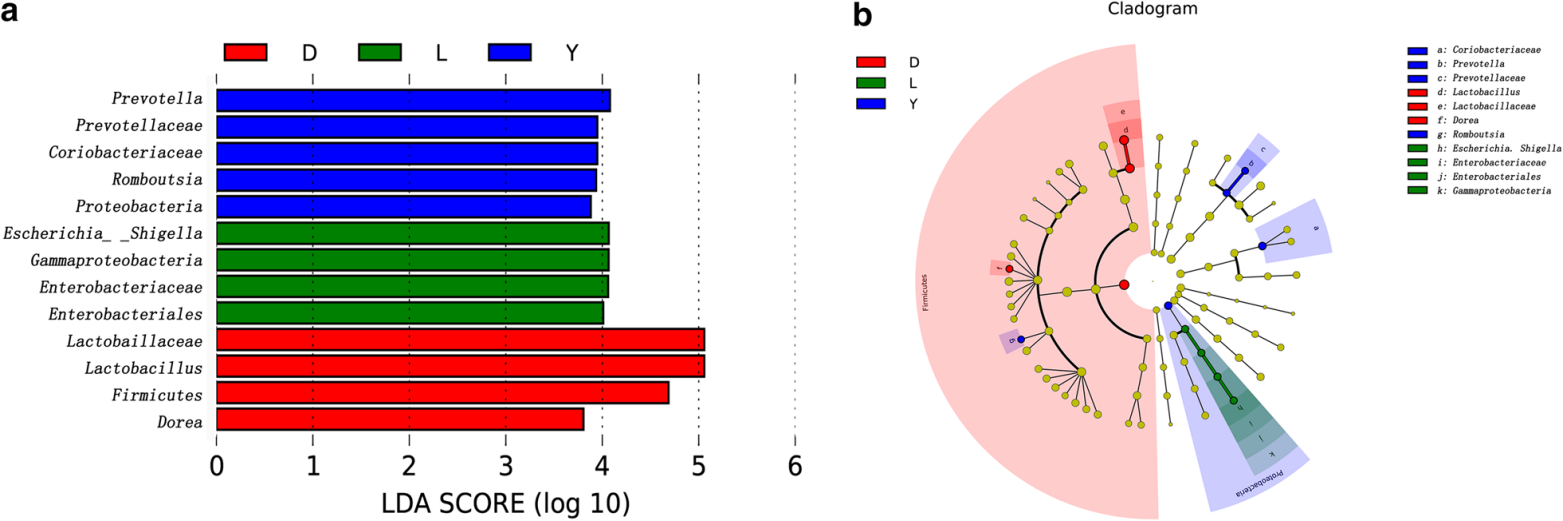
Differential abundance analysis of the bacterial taxa in the intestinal microbiomes of the three groups. **a** OTUs differentially represented at the genus level in Landrace, Duroc, and Yorkshire pigs, as identified by LEfSe. Histogram showing OTUs that were more abundant in Landrace (green color), Yorkshire (blue color) or Duroc (red color) pigs, ranked by effect size. **b** Phylogenetic tree of the microbial communities in the three groups. The phylogenetic tree with taxonomic nodes, where the diameter of the nodes indicates the relative abundance, shows the intestinal microbiota of Landrace, Duroc and Yorkshire pigs. Different groups are labeled with different colors. The red areas indicate that the species of bacteria were more abundant in Duroc pigs, the blue areas indicate that the species of bacteria were more abundant in Yorkshire pigs, and the green areas indicate that the bacteria were more abundant in Landrace pigs

Thirteen genera were shown to be significantly differentially represented among the three groups by LEfSe analysis, with 5 being more abundant in Yorkshire pigs, 4 being more abundant in Landrace pigs and 4 being more abundant in Duroc pigs. A cladogram showing the family- and genus-level abundance is shown in Fig. 3a. *Coriobacteriaceae*, *Romoboutsia* and *Prevotella* were biomarkers for Yorkshire pigs, whereas *Lactobacillus* and *Dorea* were biomarkers for Duroc pigs, and *Enterobacteriaceae* and *Gammaproteobacterta* were biomarkers for Landrace pigs. Among them, the biomarkers for the Duroc and Landrace pigs differed significantly (Fig. 3b).

**Fig. 4:**
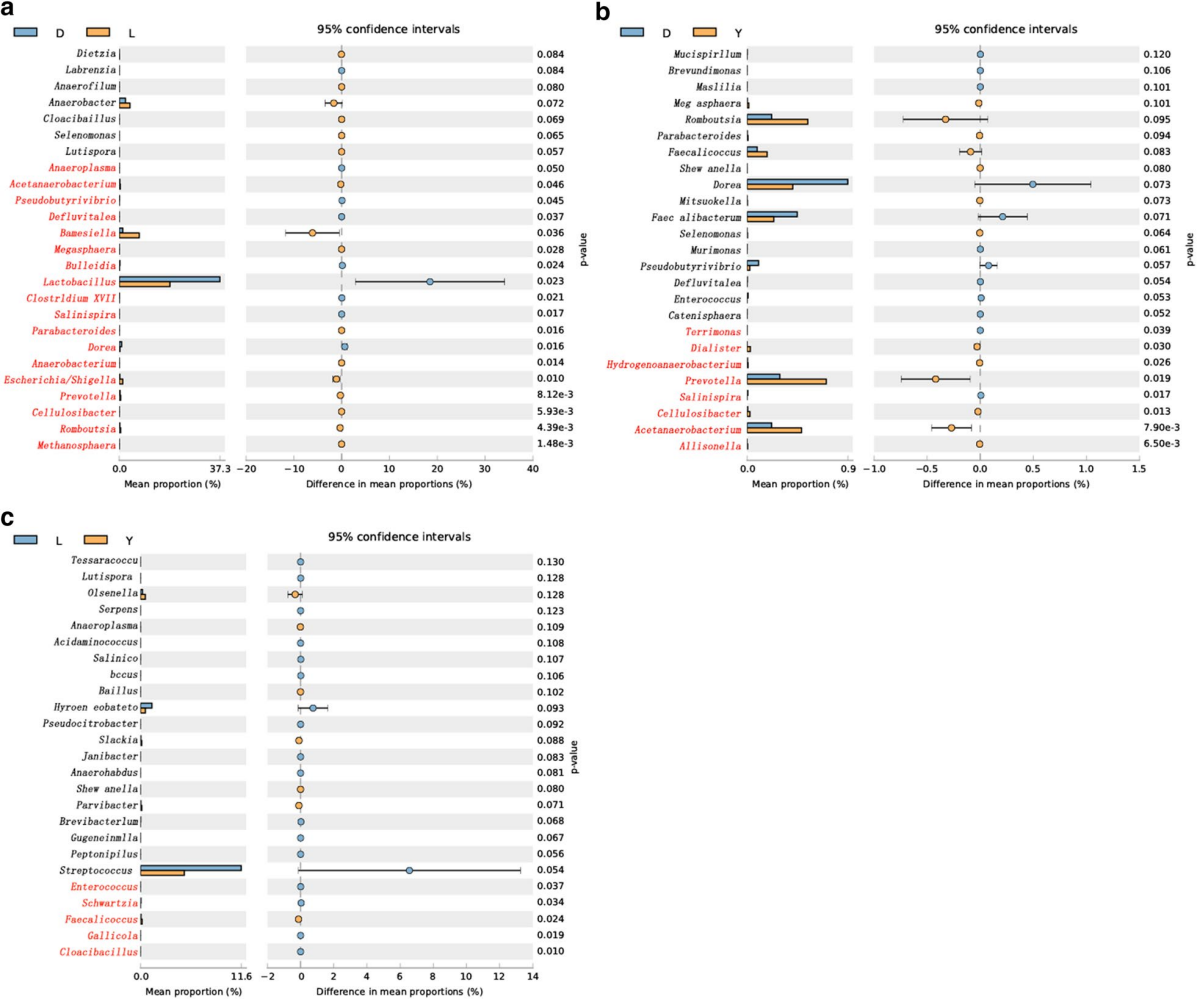
Error chart for the comparison of differences. The left part of the figure shows the abundance ratio of different microorganisms in the two groups, while the middle part shows the difference in the proportion of a classified species at the 95% confidence interval. The value on the far right is the p value, and p value < 0.05 indicates a significant difference; the species classification is marked in red. Only the 25 taxa with the lowest p values are listed. **a** Intestinal microbial difference analysis for Landrace and Duroc pigs. **b** Intestinal microbial difference analysis for Yorkshire and Duroc pigs. **c** Intestinal microbial difference analysis for Yorkshire and Landrace pigs

The error chart used to compare differences clearly shows the differences in the intestinal microbiota among different groups. The following results were drawn from the comparison of intestinal microorganisms at the genus level. The Duroc and Landrace pigs had significant differences in 18 species of gut microbes (*P* < 0.05) (Fig. 4a); the Duroc and Yorkshire pigs had significant differences in 8 species of gut microbes (*P* < 0.05) (Fig. 4b); and the Landrace and Yorkshire pigs had significant differences in 5 species of gut microbes (*P* < 0.05) (Fig. 4c). Thus, it was clear that the Duroc and Landrace pigs had the largest difference in intestinal microbiota composition, whereas the Landrace and Yorkshire pigs had the smallest difference in intestinal microbiota composition. The results showed that the levels of *Methanosphaera*, *Romboutsia*, *Cellulosibacter*, *Prevotella*, *Escherichia*, *Anaerobacterium*, *Parabacteroides*, *Megasphaera*, *Barnesiella* and *Acetanaerobacterium* in the intestinal tract of Duroc pigs were all significantly lower than those observed in Landrace pigs (*P* < 0.05); however, the levels of *Dorea*, *Salinispira*, *Clostridium*, *Lactobacillus*, *Bulleidia*, *Defluviitalea*, *Pseudobutyrivibrio* and *Anaeroplasma* in the intestinal tract of Duroc pigs were all significantly higher than those observed in Landrace pigs (*P* < 0.05) (Fig. 4a). Similarly, the levels of *Allisonella*, *Acetanaerobacterium*, *Cellulosibacter*, *Prevotella*, *Hydrogenoanaerobacterium*, *Dialister* and *Terrimonas* in the intestinal tract of Duroc pigs were all significantly lower than those observed in Yorkshire pigs (*P* < 0.05), and the level of *Salinispira* in Duroc pigs was also significantly higher than that observed in Yorkshire pigs (*P* < 0.05) (Fig. 4b). Finally, the levels of *Cloacibacillus*, *Gallicola*, *Schwartzia* and *Enterococcus* in the intestinal tract of Landrace pigs were all significantly higher than those observed in Yorkshire pigs (*P* < 0.05) (Fig. 4c and Additional file 6: Table S4).

**Fig. 5:**
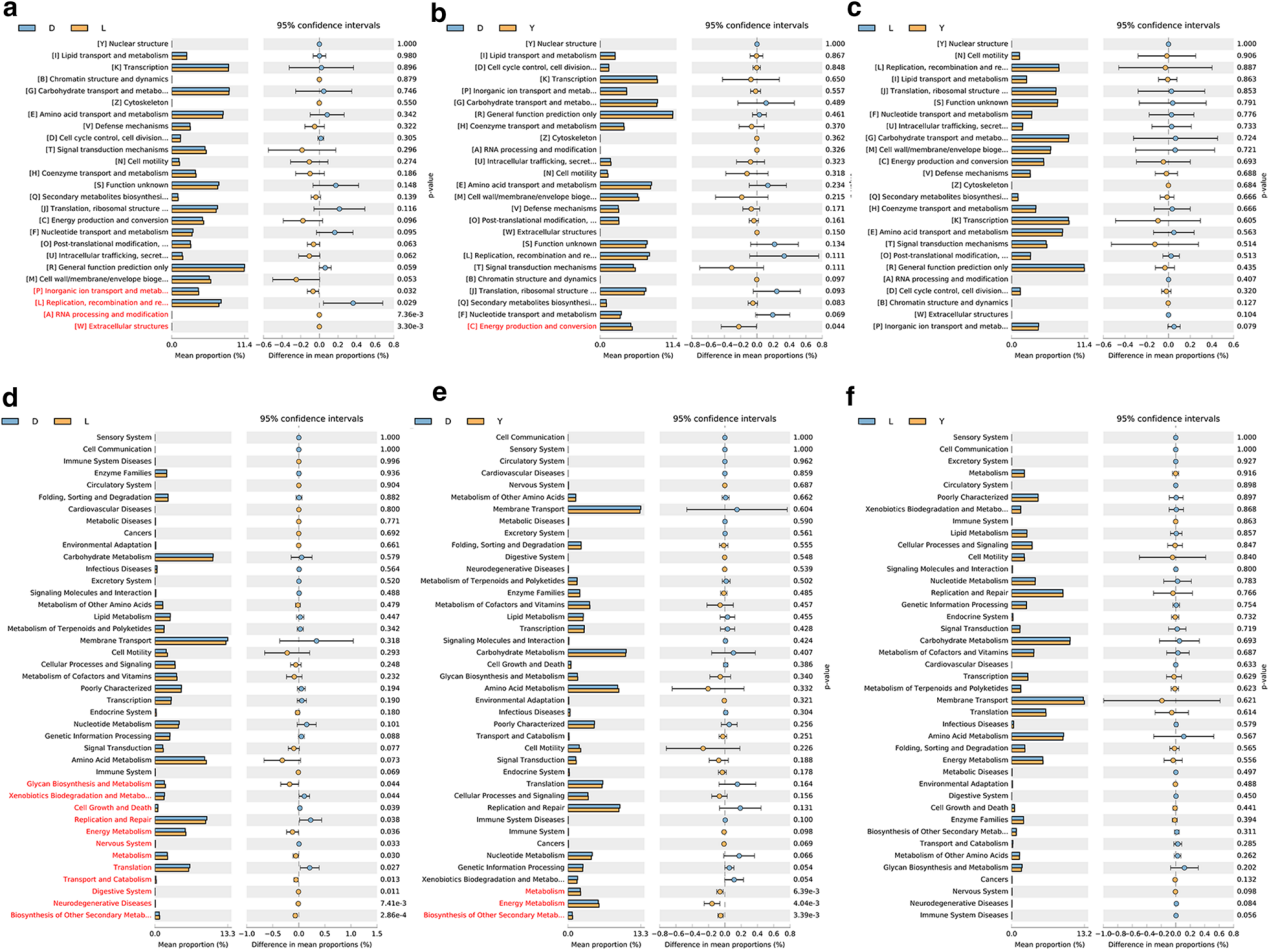
Functional prediction for differential intestinal flora in the three groups using GO and KEGG. **a** Analysis of functional differences between the intestinal flora of Landrace and Duroc pigs. **b** Analysis of functional differences between the intestinal flora of Yorkshire and Duroc pigs. **c** Analysis of functional differences between the intestinal flora of Yorkshire and Landrace pigs. **d** Analysis of KEGG pathway differences between the intestinal flora of Landrace and Duroc pigs. **e** Analysis of KEGG pathway differences between the intestinal flora of Yorkshire and Duroc pigs. **f** Analysis of KEGG pathway differences between the intestinal flora of Yorkshire and Landrace pigs. The third-level KEGG pathways are shown in the post hoc analysis plot. The significance of the differences in gene distribution between groups was determined by ANOVA with *P* < 0.05

In this study, the GO and KEGG databases were used to analyze the functions and pathways of the intestinal microflora in pigs. The enrichment of the terms extracellular structures, RNA processing and modification, inorganic ion transport, metabolism and biosynthesis of other secondary metabolites, neurodegenerative diseases, digestive system, transport and catabolism, metabolism, energy metabolism, and glycan biosynthesis and metabolism were significantly lower in Duroc pigs than in Landrace pigs (*P* < 0.05). However, the enrichment of the functions and pathways of replication, recombination and repair; translation; nervous system; replication and repair; cell growth and death; and xenobiotic biodegradation and metabolism was significantly higher in Duroc pigs than in Landrace pigs (*P* < 0.05) (Fig. 5a, d). In addition, the enrichment of the functions and pathways of energy production and conversion, biosynthesis of other secondary metabolites, energy metabolism and metabolism in Duroc pigs was significantly lower than that in Yorkshire pigs (Fig. 5b, e). Finally, it is worth noting that the intestinal flora of Yorkshire and Landrace pigs showed no significant difference in functions and pathways (*P* > 0.05) (Fig. 5c, f).

### Analysis of intestinal flora diversity of Yorkshire pigs with differences in production performance

**Fig. 6:**
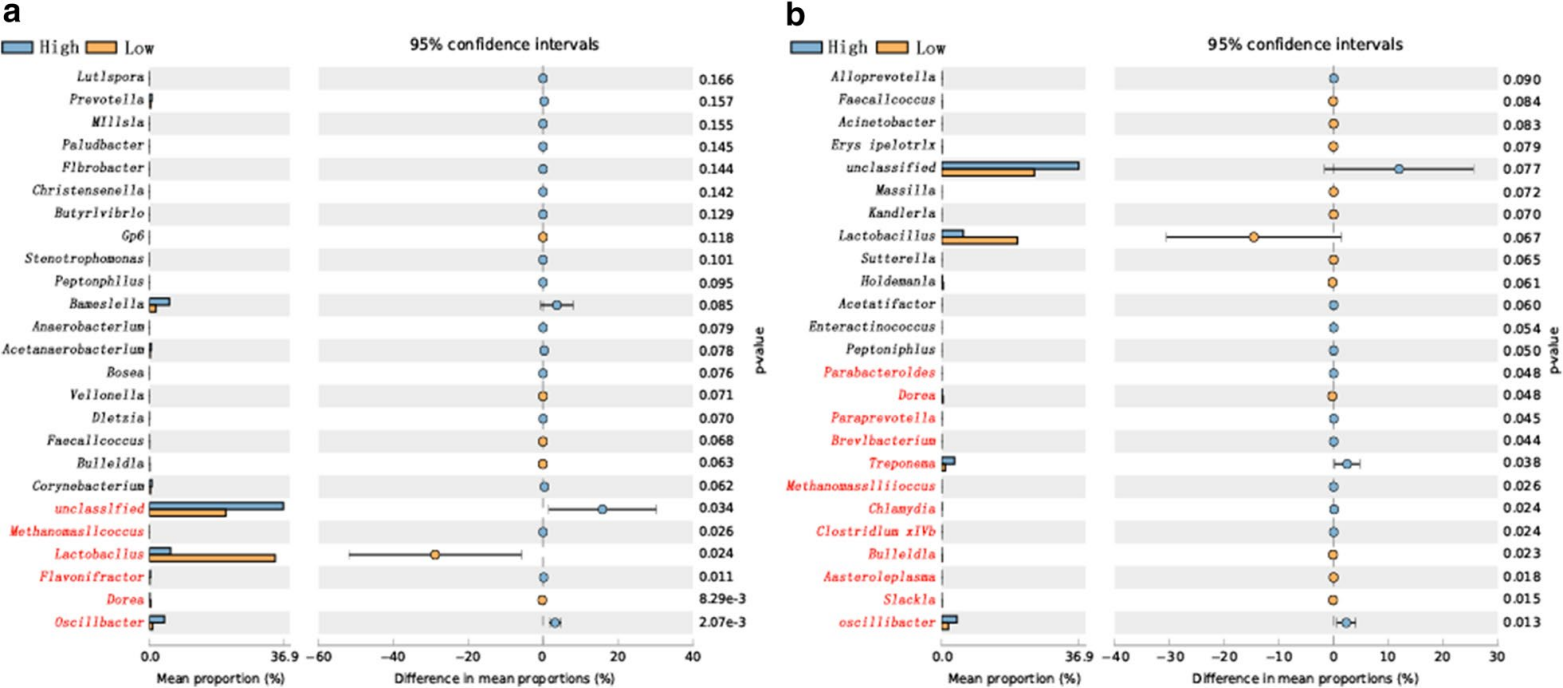
Analysis chart of intestinal flora diversity of Yorkshire pigs with significant differences in growth performance. **a** Analysis chart of intestinal flora diversity of Yorkshire pigs with significant differences in ADG. **b** Analysis chart of intestinal flora diversity of Yorkshire pigs with significant differences in FER. The left part of the figure shows the abundance ratio of different microorganisms in the two groups, and the middle part shows the difference in the proportion of classified species within the 95% confidence interval. The value on the far right is the *P* value, and *P* < 0.05 indicates a significant difference; the species classification is marked in red

In this study, the growth performance of Yorkshire pigs was first measured. After screening individuals with significant differences in production performance, the intestinal flora diversity was measured and the differences were analyzed. The different flora were compared with the different flora screened out in the previous experiment. The key bacteria that may be related to the growth performance of pigs were further screened out. First, the intestinal flora of individuals with significant differences in ADG was analyzed and the results are shown in Fig. 6a. There were 6 significantly different intestinal microbes between the high-ADG group and the low-ADG group (*P* < 0.05); the abundance of *Dorea* and *Lactobacillus* in the high-ADG group was significantly lower than that in the low ADG group (*P* < 0.05). However, the abundance of *Oscillibacter*, *Flavonifractor*, *Methanomassiliicoccus*, and *Unclassified* was significantly higher in the high-ADG group than in the low-ADG group (*P* < 0.05). Then, the intestinal flora of individuals with significantly different FER values was analyzed, and a total of 12 different flora were found (*P* < 0.05). The results showed that the proportion of *Oscillibacter*, *Clostridium XlVb*, *Chlamydia*, *Methanomassiliicoccus*, *Treponema*, *Brevibacterium* and *Paraprevotella* in the intestinal tract of the high-FER group was significantly higher than that of the low-FER group (*P* < 0.05). However, the proportions of *Slackia*, *Asteroleplasma*, *Bulleidia*, *Dorea* and *Parabacteroides* in the high-FER group were significantly lower than those in the low FER group. By comparing the results with those of previous trials, we identified five key species that may have regulatory effects on the growth performance of pigs: *Clostridium*, *Bulleidia*, *Dorea*, *Parabacteroides*, and *Lactobacillus*.

In this study, SPSS was used to analyze the correlation between the 5 candidate species and the FER, ADG, GI, and number of days taken to reach 100 kg for Yorkshire pigs. The results showed that *Lactobacillus* was significantly negatively correlated with GI and ADG, with correlation coefficients of − 0.514 and − 0.499, respectively (*P* < 0.05). *Bulleidia* was significantly negatively correlated with ADG, FER and GI, with correlation coefficients of 0.556, − 0.526, and 0.695, respectively (*P* < 0.05). *Dorea* was significantly negatively correlated with ADG and the number of days taken to reach 100 kg, with correlation coefficients of − 0.523 and − 0.436, respectively (*P* < 0.05). *Clostridium* was significantly negatively correlated with GI, with a correlation coefficient of − 0.454 (*P* < 0.05) (Table 1).

**Table 1:** Correlation analysis between key flora and pig growth performance

Item	*Lactobacillus*	*Parabacteroides*	*Dorea*	*Bulleidia*	*Clostridium*
ADG	− 0.499^a^	0.114	− 0.523^a^	0.556^a^	− 0.220
FER	0.159	− 0.221	0.038	− 0.526^a^	0.271
GI	− 0.514^a^	0.216	− 0.350	0.695^a^	− 0.454^a^
Days taken to reach 100 kg	0.378	− 0.091	0.436^a^	− 0.420	0.089

^a^Significant correlation (*P* < *0.05*), while the absence of * indicates nonsignificant correlation (*P* > 0.05)

### Effect of cell level on the functions of differentially abundant flora

**Fig. 7:**
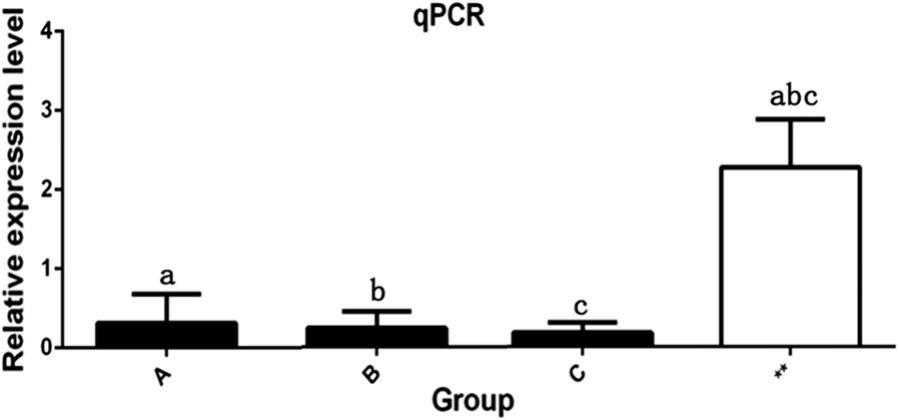
Bar chart of qPCR results. **a** *Lactobacillus* treatment at 1 × 10^6^ CFU, **b** *Lactobacillus* treatment at 1 × 10^7^ CFU, **c** *Lactobacillus* treatment at 1 × 10^8^ CFU; ** represents the blank treatment control group

*Lactobacillus* is a relatively common bacterium, and in this study, *Lactobacillus* was identified as a key bacterium that may affect the growth performance of pigs, *Lactobacillus* was selected for functional exploration. After 12 h of intestinal epithelial cell treatment with *Lactobacillus*, qPCR was used to identify MC4R gene expression in the experimental group and the control group. The results showed that the lactic acid bacteria in the groups containing 1 × 10^6^, 1 × 10^7^ and 1 × 10^8^ CFU inhibited the expression of the MC4R gene, making the expression level of the MC4R gene significantly lower than that in the control group (*P* < 0.05). With the increase in *Lactobacillus* concentration, its inhibitory effect on MC4R gene expression gradually increased (Fig. 7).

## Discussion

There are a large number of microorganisms present in the intestine, and a large number of studies have shown that the intestinal flora has a significant regulatory effect on the growth performance of animals. In this study, a similar phenomenon was found by analyzing the relationship between the growth performance of pigs and intestinal microorganisms.

The results of this study showed that regardless of the genetic background, the intestinal microbiota was composed of *Firmicutes* and *Bacteroidetes* at the phylum level, but the abundances and proportions of *Firmicutes* and *Bacteroidetes* in the intestinal tract of pigs of the different genetic backgrounds were different. Previous studies have suggested that genetic effects was significantly correlated with microbiome composition (Hildebrand et al. 2013). Furthermore, other studies have shown that the pig breed affects the composition of *Firmicutes*, *Bacteroidetes*, and sulfate-reducing bacteria, which are more abundant in Chinese native pig breeds than in foreign breeds (Yang et al. 2014). *Firmicutes* and *Bacteroidetes* are also the two most abundant phyla in the healthy human gut microbiota, but the ratio of these two phyla varies among individuals (Zhang et al. 2015). A previous study reported similar results for the gastro intestinal tract of other breeds of pigs (Kim et al. 2016). Subsequently, to eliminate the influence of genetic background on the results, we studied the intestinal flora composition of Yorkshire pigs with differences in growth performance and obtained similar results. Another study examined the relationship between the composition of gut microbes and growth rates and fat accumulation and observed that *Sphingobacteria* in the phylum *Bacteroidetes* and *Deltaproteobacteria* in the phylum *Proteobacteria* were abundant in the gut, promoting fat production in some animals (Yang et al. 2016; Zhao et al. 2015). *Bacteroidetes* and *Proteobacteria* also play an important role in the growth performance of pigs (Yang et al. 2016), similarly, we also found that the levels of *Bacteroidetes* and *Proteobacteria* were significantly different in the intestines of pigs with different growth performances. In addition, the genus level results observed in this study indicated that *Lactobacillus*, *Streptococcus*, *Lachnospiracea* and *Barnesiella* are the core bacterial genera in pig intestines. Previous studies have reported that *Prevotella* and *Streptococcus* are the most abundant bacterial genera in the intestines of pigs (Ramayo-Caldas et al. 2016), which is somewhat different from the results of this study. Furthermore, another study showed that the abundance of *Prevotella* decreased from 30% to 4.0% of total bacteria in the gut of aged pigs, which is related to the gradual increase in the intestinal digestion and absorption capacity of pigs (Kim et al. 2016). Therefore, it was speculated that the increasing intestinal digestive capacity of pigs led to a gradual decrease in the proportion of *Prevotella* in the intestines of pigs, while *Lactobacillus* contributed to the increasing intestinal digestive capacity, leading to the high accumulation of *Lactobacillus* in the intestines of pigs.

We found that the intestinal microbiota compositions of Duroc and Landrace pigs were significantly different, primarily with respect to the abundance of *Anaeroplasma*, *Acetanaerobacterium*, *Pseudobutyrivibrio*, *Defluvitalea*, *Barnesiella*, *Megasphaera*, *Bulleidia*, *Clostridium*, *Salinispira*, *Parabacteroides*, *Dorea*, *Anaerobacterium*, *Escherichia*, *Prevotella*, *Cellulosibacter*, *Romboutsia*, *Lactobacillus* and *Methanosphaera*. Similarly, our functional predictions have shown that differences in intestinal flora can lead to differences in function related to the growth performance of pigs. Subsequently, we analyzed the intestinal flora of Yorkshire pigs with different growth properties, and the results showed that *Dorea*, *Lactobacillus*, *Bulleidia*, *Clostridium*, and *Parabacteroides* were again identified as being significantly different. Therefore, the above bacterial groups were preliminarily considered to be related to the growth performance of pigs. The subsequent correlation analysis between the growth performance of pigs and the key flora showed that the above flora were indeed the key flora regulating the growth performance of pigs. Some studies have come to similar conclusions, showing that *Methanosphaera*, *Prevotella* and *Romboutsia* are linked to fat accumulation (Guo et al. 2018); it has been shown that *Escherichia*/*Shigella*, *Parabacteroides* and *Megasphaera* have a specific correlation with the growth performance of pigs (Yin et al. 2018). This suggests that the intestinal flora does have an effect on pig performance, but the exact mechanism is unclear. Notably, *Salinispira*, a bacterium unique to the Duroc gut, was identified in this study. However, because there are currently no reports on the function of *Salinispira* in the intestinal tracts of pigs, further research on this bacterium is required.

Previous studies revealed that many species of *Escherichia*-*Shigella* and *Romboutsia*, which are most abundant in the intestinal tract, contribute to the degradation of glucose and fructo-oligosaccharides (Delgado-Andrade et al. 2017; Gerritsen et al. 2017). In this study, the intestinal abundance of these two bacterial groups was also found differ significantly between Duroc and Landrace pigs, but there was no significant difference in the levels of *Escherichia/Shigella* and *Romboutsia* in the intestines of Yorkshire pigs with differences in growth performance. This suggests that the levels of these two types of gut flora associated with fat accumulation in pigs may be influenced by the genetic background. Many species of *Lactobacillus* and *Streptococcus* (the prevalent genera in the colon) contribute to lactic acid production (Matato et al. 2017).

Another study suggested that the enzymatic digestion and absorption of starch constitute the predominant functions of the small intestine, while the large intestine primarily ferments nonstarch polysaccharides via bacteria and produces SCFAs, which serve as important nutrients for the epithelium and body tissues (Serena et al. 2008). *Lactobacillus* plays a key role in this process, indicating that this genus has an impact on the growth performance of pigs. In this study, it was found that at the genus level, *Lactobacillus* had the highest proportion in the intestinal tract, and the levels of *Lactobacillus* in the intestinal tracts of pigs with differences in growth performance were significantly different. In addition, there is a negative correlation between the growth performance of pigs and the content of *Lactobacillus* in the intestines. The higher the content of *Lactobacillus* is, the lower the growth performance of pigs. This conclusion is consistent with the results of the two experiments in this study. The OTUs associated with *Streptococcus* were associated with lactic acid-producing bacteria, and *Escherichia*/*Shigella* and *Romboutsia* were associated with glucose degradation and absorption (Delgado-Andrade et al., 2017; Gerritsen et al., 2017). This conclusion is similar to the results of this study and to some extent supports the results of this study. In addition, this study also found that the greater the difference in pig growth performance is, the greater the number of significantly different flora in the pig intestines. However, not all differences in gut flora affect function. Many members of these families show a high potential for fermenting various polysaccharides and dietary proteins (Meehan et al. 2014; Su et al. 2014).

In summary, the intestinal flora of Duroc, Landrace and Yorkshire pigs were identified via 16S rRNA sequencing in this study. Through functional prediction of the intestinal flora of different pig breeds, the differences in intestinal flora among the different pig breeds were shown to potentially lead to differences in the growth performance of the pigs, and these results were also verified to some extent by the phenotype determination conducted at the beginning of the study. Moreover, we analyzed the correlation between the flora and growth performance in Yorkshire populations and finally screened *Lactobacillus*, *Barnesiella*, *Clostridium* and *Dorea,* which were significantly related to the growth performance of pigs. On the other hand, this study elucidated the effect of *Lactobacillus* on MC4R gene expression in pig intestinal epithelial cells, providing some references for studying the influence of flora on host phenotypes. These findings can enhance our understanding of the relationship between the intestinal flora and the growth performance of pigs and provide a theoretical basis for subsequent studies on the regulation of host growth performance by the intestinal microflora.

## Supplementary information

**Additional file 1: Table S1.** Growth characteristics and body size data of different breeds.

**Additional file 2: Table S2.** Diversity indices and summary of the 16S rRNA gene pyrosequencing data.

**Additional file 3: Table S3.** Phylum level intestinal microbiota difference analysis results for the different breeds.

**Additional file 4: Figure S1.** PCoA based on unweighted UniFrac distances. Each point represents a sample.

**Additional file 5: Figure S2.** Venn diagram analysis of the OTUs among the different pig breeds.

**Additional file 6: Table S4.** Genus level intestinal microbiota difference analysis results for the different pig breeds.

## Data Availability

The raw sequencing data in this study were deposited in the NCBI Sequence Read Archive (SRA) under accession number SUB6206792.

## Abbreviations

rRNA: Ribosomal RNA
ADG: Average daily gain
FER: Feed efficiency ratio
GI: Growth index
BW: Average body weight
BL: Body length
BH: Body height
CG: Chest girth
RG: Rump girth
TG: Tube girth
BT: Backfat thickness
PCRs: Polymerase chain reactions
ACE: Abundance-based coverage estimation
PCoA: Principal coordinate analysis
LEfSe: Linear discriminant analysis effect size
KW: Kruskal–Wallis
KEGG: Kyoto Encyclopedia of Genes and Genomes
GO: Gene ontology
