# Supplementary figures and images for: Identification of microflora related to growth performance in pigs based on 16S rRNA sequence analyses
Source: AMB Express. 2020 Oct 29;10:192. doi: 10.1186/s13568-020-01130-3 (PMC7596147; doi:10.1186/s13568-020-01130-3)

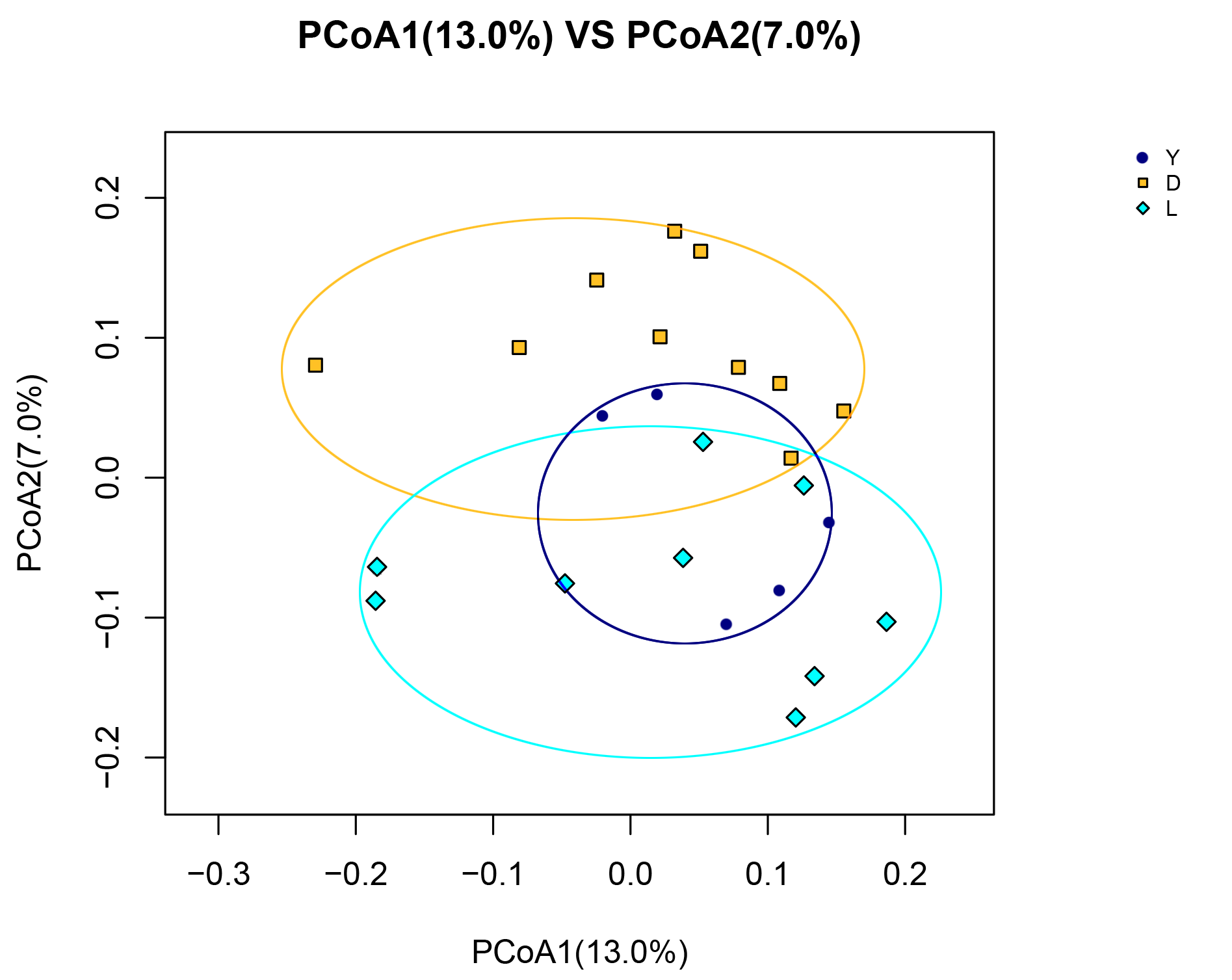

Supplement: Supplementary file 4 — Additional file 4: Figure S1. PCoA based on unweighted UniFrac distances. Each point represents a sample. [file 13568_2020_1130_MOESM4_ESM.png]

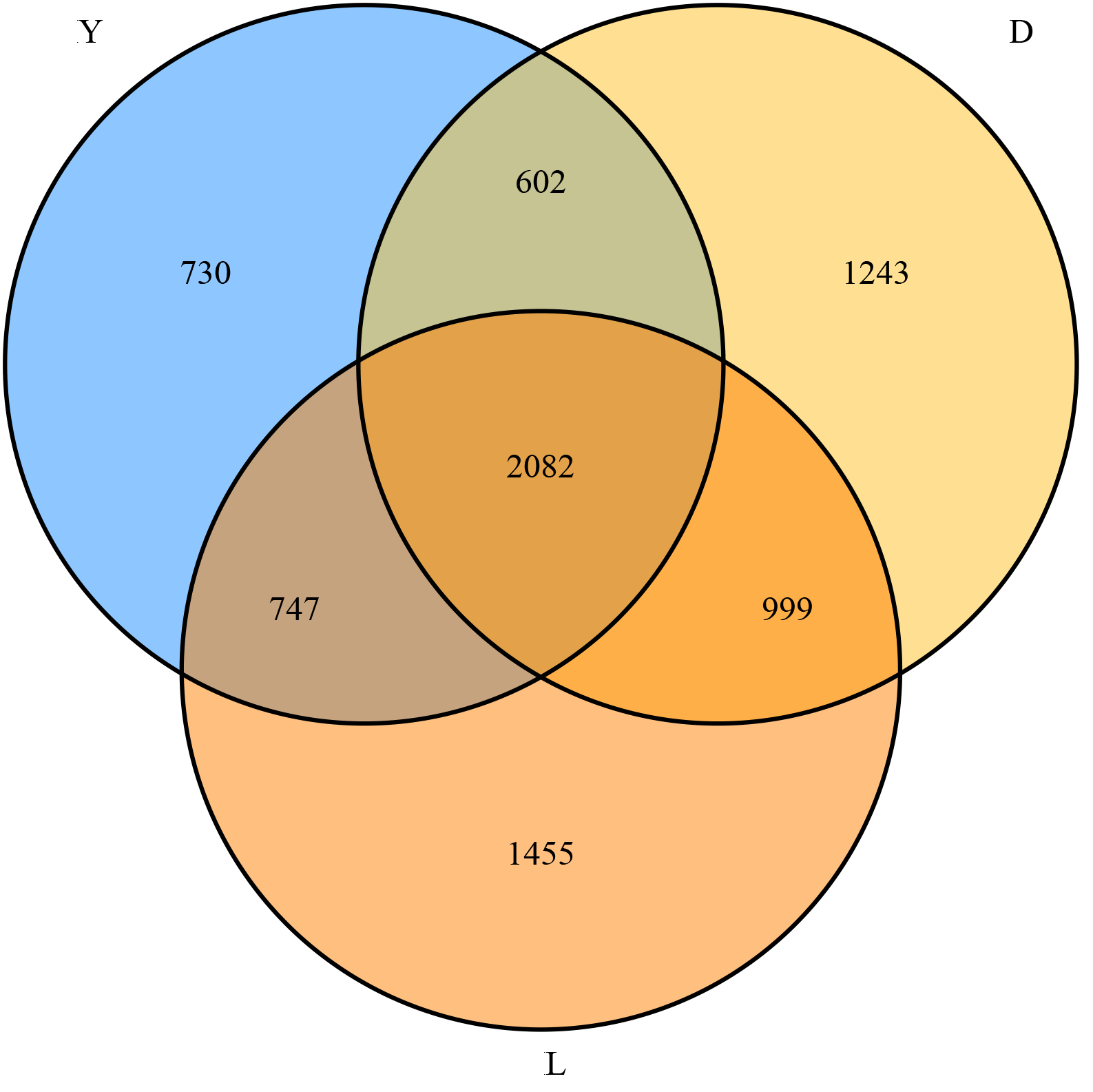

Supplement: Supplementary file 5 — Additional file 5: Figure S2. Venn diagram analysis of the OTUs among the different pig breeds. [file 13568_2020_1130_MOESM5_ESM.png]
